# Persona-prompted LLM agents achieve modest but genuine prediction of human social media reactions

**DOI:** 10.1038/s41598-026-66277-8

**Published:** 2026-09-03

**Authors:** Ljubiša Bojić, Alexander Felfernig, Bojana Dinić, Velibor Ilić, Achim Rettinger, Vera Mevorah, Damian Trilling

**Affiliations:** 1 Institute for Artificial Intelligence Research and Development of Serbia, 1 Frukogorska 1, Novi Sad, 21000 Serbia; 2 Institute for Philosophy and Social Theory, Digital Society Lab, University of Belgrade, Natalije 45, Belgrade, 11000 Serbia; 3 Complexity Science Hub, Metternichgasse 8, Vienna, 1030 Austria; 4 Graz University of Technology, Inffeldgasse 16b/2, Graz, Austria; 5 Faculty of Philosophy, University of Novi Sad, Dr Zorana Đinđića 2, Novi Sad, 21102 Serbia; 6 University of Trier, Universitätsring 15, 54296 Trier, Germany; 7 Vrije University Amsterdam, De Boelelaan 1105, Amsterdam, 1081 HV Netherlands

**Keywords:** LLM-based social simulation, AI agents, Social media behavior prediction, Persona prompting, Behavioral benchmarking, Mathematics and computing, Psychology, Psychology

## Abstract

Social media platforms mediate how billions form opinions and engage with public discourse. As autonomous AI agents increasingly participate in these spaces, understanding their behavioral fidelity becomes critical for platform governance and democratic resilience. Previous work demonstrates that LLM-powered agents can replicate aggregate survey responses, yet few studies test whether agents can predict specific individuals’ reactions to specific content. This study benchmarks LLM-based agents’ accuracy in predicting human social media reactions (like, dislike, comment, share, no reaction) across 120,000 + unique agent-persona combinations derived from 1,511 Serbian participants and 27 large language models. In Study 1, agents achieved 70.7% overall accuracy, with LLM choice producing a 13%-point performance spread. Study 2 employed binary forced-choice (like/dislike) evaluation with chance-corrected metrics. Agents achieved Matthews Correlation Coefficient (MCC) of 0.29, indicating genuine predictive signal beyond chance. However, conventional text-based supervised classifiers using TF-IDF representations outperformed LLM agents (MCC of 0.36), indicating that the predictive signal derives from semantic text content rather than from any capacity for individualized behavioral simulation. The genuine but modest predictive validity of zero-shot persona-prompted agents suggests that, while current accuracy is insufficient for precise individual targeting, the capacity to predict reactions at rates above chance warrants attention in discussions of AI-driven influence and social simulation methodology. The advantage of zero‑shot agents is that they require no task‑specific training, which makes large‑scale deployment easy across diverse contexts, including election campaigns and mass‑scale manipulation. Limitations include single-country sampling. Future research should explore multilingual testing and fine-tuning approaches.

## Introduction

The idea that large language models (LLMs) can act as proxies for human participants in social and behavioral research has moved within a few years from a speculative proposition to a rapidly expanding research program. Park^1^ placed 25 LLM-powered generative agents in a sandbox environment and found that they organized social events, formed relationships, and coordinated daily routines in ways that human observers judged believable, while foundation models trained on large-scale behavioral data have since achieved human-level prediction of cognition across diverse paradigms^2^. That early proof of concept has given way to more ambitious projects. Park^3^ grounded generative agents in two-hour interviews with 1,052 individuals and reproduced their General Social Survey responses at 85% of the participants’ own two-week test-retest accuracy, and Yang et al.^4^ scaled the approach to one million agents in the OASIS platform, simulating information spread, group polarization, and herd effects on Reddit-like and X-like platforms. Together these advances suggest that LLM-based social simulation could become a general-purpose tool for testing policy interventions, modelling opinion dynamics, and stress-testing platform design where real-user experiments would be impractical or unethical^5^.

In a complementary line of work, Altera.AL^6^ introduced Project Sid, demonstrating that 10 to over 1,000 LLM-powered agents placed in a Minecraft environment could autonomously develop specialized professional roles, adhere to and modify collective rules through democratic processes, and engage in cultural and religious transmission across multiple simulated societies. This suggests that emergent social organization is achievable even without explicit programming of societal structures.

The predictability of human behavior from digital traces has been studied for over a decade. Kosinski et al.^7^ demonstrated that Facebook Likes alone could predict sensitive personal attributes with high accuracy. More recently, Derner et al.^8^ showed that ChatGPT can infer Big Five personality traits from short texts with accuracy comparable to human raters, though they documented a “positivity bias” in model outputs.

Yet the empirical foundations of this research program remain thin, and the gap between demonstrated capability and validated reliability is large. Much of the existing work has evaluated LLM agents on their ability to reproduce aggregate survey distributions or to generate text that human raters judge as believable, but relatively few studies have asked whether LLM agents can accurately predict the specific behavioral reactions of specific kinds of people to specific kinds of content (see also^9^. This distinction matters. Aggregate accuracy can mask systematic failures at the level of subgroups, content types, or persona configurations, and such failures would severely limit the usefulness of LLM-based simulation for any application in which fine-grained behavioral prediction is the goal.

The question of how faithfully LLM agents can embody assigned personas sits at the center of this concern. The dominant conditioning method is persona prompting, in which a textual description of the agent’s demographic, attitudinal, or psychological profile is prepended to the model’s input^10,11^. Argyle^10^ showed that conditioning GPT-3 on demographic backstories from the American National Election Studies produced opinion distributions that tracked human subgroup responses, a result they called “silicon sampling,” and Törnberg et al.^11^ populated a simulated platform with 500 persona-based agents to show that a bridging feed algorithm promoted more cross-partisan dialogue than conventional designs. Later work tempers this optimism. Hu et al.^12^ found that persona variables explained less than 10% of the variance in human annotations across most tasks, with the modest gains concentrated in datasets of intermediate annotator disagreement, and Liu et al.^13^ reported that agents steered toward incongruous personas, such as political liberals who support higher military spending, were 9.7% less accurate than those given congruous ones and often defaulted to the stereotypical stance of the demographic category. The relationship between persona description and agent behavior may therefore be more fragile than the simulation literature has assumed.

A related and even more fundamental criticism holds that the apparent human-likeness of LLM outputs may be an artifact of the models’ training data rather than evidence of any capacity for behavioral reasoning. Dillion et al.^14^ argued that LLMs may have encountered the answers to many psychological and behavioral tasks during pre-training and can therefore reproduce correct responses through memorization rather than through genuine understanding of the constructs involved. Qi^15^ and Wang et al.^16^ offered similar critiques, noting that LLMs sometimes produce correct answers followed by incorrect explanations, a pattern consistent with surface-level recall rather than deep comprehension. Cui^17^ attempted to address this critique by testing GPT-3.5-turbo on a personality-behavior association, the link between MBTI types and imposter phenomenon scores, that was published after the model’s September 2021 training cutoff. Their results showed 75% consistency between GPT and human responses at the trait level and 69% at the type level, providing some evidence that GPT’s human-likeness extends beyond memorization. Still, the study tested only a single behavioral outcome and used a comparatively old model, leaving open the question of whether these results generalize across diverse behavioral domains and more capable model families.

A distinct strand goes further, questioning whether LLM outputs reflect genuine reasoning at all. Mahowald^18^ distinguished between formal linguistic competence (producing grammatically correct language) and functional linguistic competence (using language to accomplish goals in the world), arguing that LLMs excel at the former but show inconsistent performance on the latter. Loru^19^ found that LLMs may rely on lexical associations and statistical priors rather than contextual reasoning when performing evaluative judgments, suggesting that apparent reasoning reflects surface-level pattern matching. Suzgun^20^ demonstrated that LLMs systematically fail to distinguish belief from knowledge and fact, particularly in first-person contexts.

Zewail et al.^21^ showed that LLMs stereotype the moral values of non-Western populations in predictable ways, overemphasizing concerns common in Western societies. Guilbeault et al.^22^ demonstrated that online media systematically represents women as younger than men across occupations and social roles, and that LLMs trained on this content internalize and reproduce these distortions. Because LLMs derive their representations from biased social data, such stereotyping may be an inherent feature of current architectures rather than a correctable flaw. These findings raise the possibility that behavioral prediction by LLM agents may reflect access to rich but biased textual representations rather than genuine agentic simulation, a possibility that the present study directly tests through comparison with conventional text-based classifiers.

The practical implications of these LLM capabilities are substantial, for example in studying algorithmic polarization^11,23^ and misinformation spread^4^. Recent work by Bojić et al.^24^ introduced an LLM-driven agent-based simulation showing that higher levels of recommender-system personalization can substantially amplify affective and structural polarization, which further demonstrates the relevance of LLM agents for studying algorithmic effects on social media ecosystems. These capabilities extend beyond social media as well, with multimodal large language models proposed to integrate diverse data streams in sixth-generation wireless networks, an illustration of the expanding scope of LLM deployment across digital infrastructure^25^.

Quattrociocchi and colleagues recently demonstrated that injecting AI-generated users with pro-social briefings into a simulated social network could reduce echo chamber dynamics, a finding that attracted widespread media attention and that illustrates the potential of LLM-based simulation for platform governance research^26^. Wu et al.^27^ proposed a pragmatic framework for determining when LLM-based social simulations can meaningfully advance scientific understanding, arguing that these simulations are most defensible when used to explore collective patterns rather than individual decision trajectories, and when validation methods are carefully matched to the research objectives and simulation scale. Piao^28^ developed AgentSociety, a framework that adopts various validation strategies comparing simulated outputs with real-world social indicators. These developments collectively point toward a maturing field, but one that still lacks systematic benchmarks for the accuracy with which individual agents react to specific stimuli.

The role of persona specificity in shaping agent behavior has received little systematic attention despite its centrality to the simulation enterprise. Most studies compare persona-conditioned agents against no-persona baselines without varying the level of detail in the description, so whether richer profiles yield more accurate predictions remains open, and the few relevant results are suggestive rather than conclusive. Work on MBTI-based simulation found that agents whose initial personality setup failed required additional trait descriptions to reach the correct profile, which hints that more detail improves alignment^17^. Serapio-García^29^ found that LLMs could simulate Big Five traits but produced unrealistically large correlations between dimensions, which suggests a coarse rather than fine-grained representation of personality. Bodroža et al.^30^ reported that models defaulted to a socially desirable and prosocial profile, with higher agreeableness and conscientiousness and lower Machiavellianism, an idealized rather than human-typical structure. Peters and Matz^31^ showed that GPT-4 could infer Big Five traits from Facebook status updates at accuracy comparable to supervised machine learning, though with heterogeneity across age and gender. Cloud^32^ added a striking result, showing that student models trained on semantically unrelated data such as number sequences generated by a teacher model with a given trait acquired that trait through what they termed subliminal learning, which implies that LLMs encode behavioral dispositions well beyond explicit persona descriptions and complicates simple assumptions about how persona input maps to behavioral output.

An additional gap in the literature concerns the role of content type in moderating agent accuracy. Social media platforms host a heterogeneous mix of content categories, from hard news and political commentary to entertainment, humor, and personal updates, and there is reason to expect that LLM agents will differ in their ability to predict human reactions across these categories. Bojić et al.^33^ showed that GPT-4 outperformed human participants in interpreting linguistic pragmatics, suggesting strong performance on tasks that require contextual inference. Yet Cheng et al.^34^ found that LLMs tend to produce caricatured rather than authentic representations of social identities, and this tendency may be more pronounced for some content types (e.g., politically charged news) than for others (e.g., entertainment). To date, no study has systematically examined whether LLM agent accuracy varies by post type on social media, despite the obvious relevance of this question for any simulation that aims to replicate the diversity of online discourse.

The question of what happens when a persona description is mismatched with or irrelevant to the content being evaluated is equally understudied. In real social media environments, users encounter posts that fall outside their areas of expertise, interest, or demographic relevance, and their reactions in such cases may differ systematically from their reactions to content that aligns with their identity and experience. If LLM agents are more accurate when the persona matches the content domain and less accurate when it does not, this would have direct implications for how personas should be constructed and deployed in simulation studies. The existing literature offers indirect evidence that mismatch matters: Liu et al.^13^ showed that incongruous persona-stance pairings reduce steerability, and La Cava and Tagarelli ^35^ found that open-source LLMs varied in their ability to maintain persona consistency across different task contexts. But no study has directly tested whether persona-content mismatch degrades reaction prediction accuracy in a social media simulation setting.

A methodological concern in this literature is the choice of baseline against which agent performance is evaluated. Many studies report accuracy relative to random chance (e.g., 50% for binary classification), but this baseline fails to account for class imbalance in human responses. If 70% of humans like a post, a trivial strategy of always predicting “like” achieves 70% accuracy without any genuine prediction. Standard practice for imbalanced classification recommends chance-corrected metrics such as Matthews Correlation Coefficient (MCC), which equals zero for any strategy that ignores the input, and minority-class lift, which measures improvement over base-rate guessing for underrepresented behaviors. The present study follows this practice by evaluating agent performance against multiple baselines and reporting chance-corrected metrics alongside raw accuracy.

### The present study

The present study addresses these gaps through a large-scale benchmarking simulation that tests whether persona-prompted LLM agents can predict individual human reactions to specific social media posts, and whether they do so more accurately than conventional supervised text classifiers given the same information. Comparisons across persona specificity, model, and content conditions map where this predictive accuracy holds and where it breaks down, and the guiding question is whether persona-driven agents are ready to serve as general proxies for human social media users or whether their utility is bounded by persona quality, content domain, and the limits of LLM behavioral modelling. Reactions are operationalized as the options most common on social media, like or dislike, comment, share, and no reaction, a measure that is ecologically valid, since it reflects how billions of users engage with content, and fine-grained enough to capture differences beyond simple engagement. Raw accuracy is the proportion of correct matches between human and agent reactions to the same posts. Because these reactions are class-imbalanced, the study emphasizes chance-corrected metrics, including MCC, balanced accuracy, and minority-class lift, alongside raw accuracy.

Because prior theory offers limited guidance on how agent prediction accuracy should vary across content conditions, several of the analyses that follow are best understood as exploratory benchmarking comparisons rather than strictly confirmatory theory tests, intended to establish empirical reference points for future work.

First, we hypothesized that LLM agents would demonstrate genuine predictive signal for human reactions, operationalized as achieving a Matthews Correlation Coefficient (MCC) significantly greater than zero (**H1**). An MCC significantly above zero indicates that the agents capture meaningful covariation between persona–post pairs and human reactions beyond what the baselines capture.

Second, we assumed that an increase in an agent’s persona (i.e., information provided in prompts) description would improve accuracy (**H2**). This directly tests the assumption, widespread in the simulation literature but rarely validated, that richer persona information produces more human-like agent behavior.

Third, we assumed that mismatched or irrelevant persona descriptions would reduce reaction accuracy compared with matched-related descriptions (**H3**). This question addresses the robustness of persona-based agents under conditions of persona-content misalignment, a scenario that is common in real social media use but absent from most simulation studies.

Fourth, we explored whether prediction accuracy differed between entertainment/lifestyle and news/politics posts. Although there are reasons to expect lower predictability for news/politics content, this comparison was treated primarily as an exploratory benchmarking test of domain-specific variation in agent accuracy (**H4**). This question tests whether the capacity of agents to predict human reactions is general or domain-specific, a distinction with direct implications for the external validity of social media simulations.

Fifth, we examined whether prediction accuracy differed between positively and negatively valenced posts. This comparison was likewise treated as an exploratory benchmarking analysis, intended to assess whether emotional framing systematically affects the ability of LLM agents to approximate human reactions (**H5**). This hypothesis examines whether the emotional valence of a post influences the ability of agents to approximate human reactions.

Sixth, we hypothesized that LLM-based agents would outperform conventional supervised machine-learning benchmarks in predicting individual reactions, given their capacity for contextual reasoning and persona simulation (**H6**). This hypothesis tests whether agent-based simulation provides unique predictive advantages beyond what standard feature-based classifiers can achieve when given access to the same semantic information.

## Methodology

**Fig. 1:**
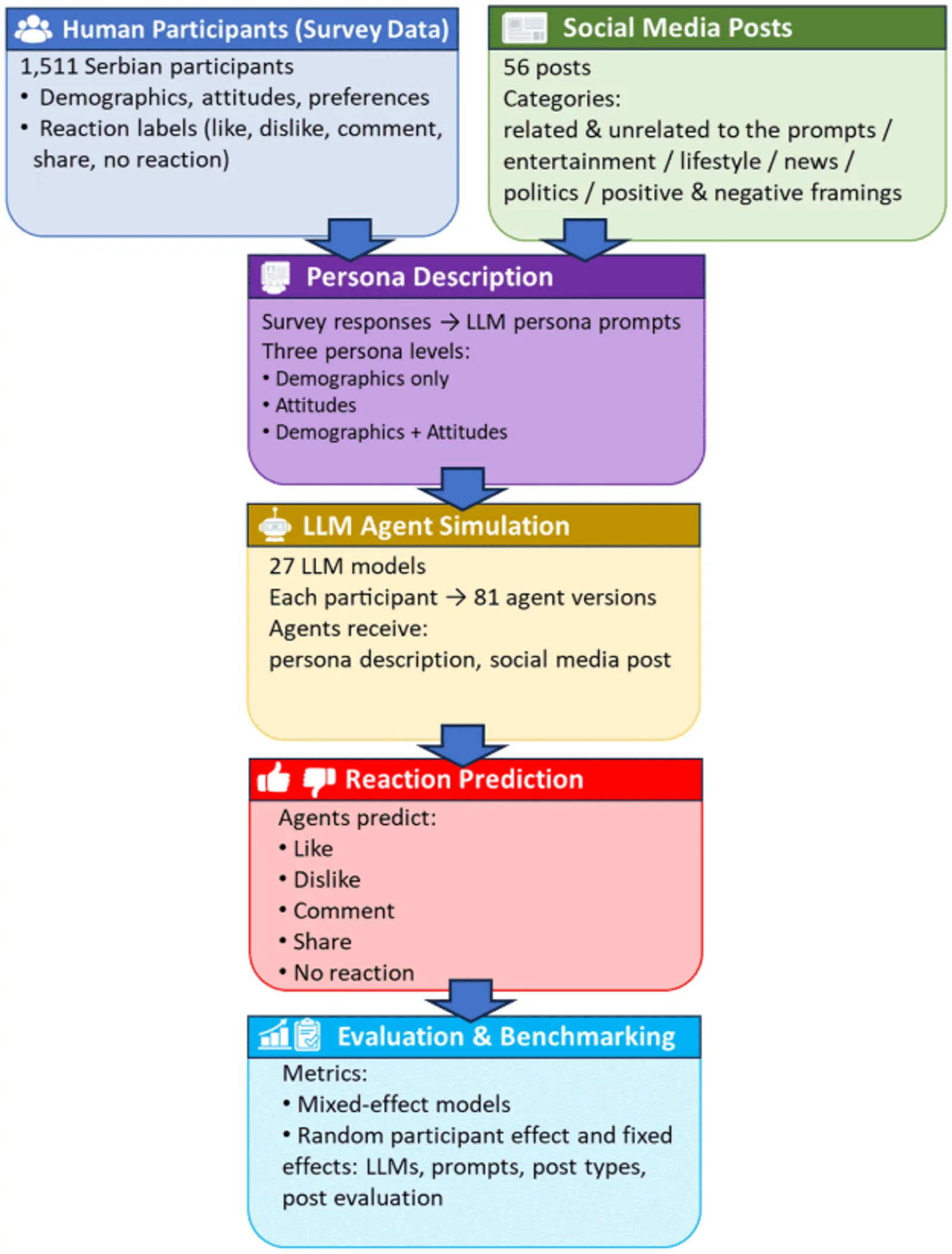
Schematic overview of the study design. Survey responses from 1,511 Serbian participants were translated into persona descriptions at three specificity levels and paired with 27 large language models to generate agents, which predicted each participant’s reactions (like, dislike, comment, share, no reaction) to the same 56 posts the participant evaluated. Agent-human agreement was assessed with mixed-effects models treating LLM, prompt type, post type, and valence as fixed effects.

Human participants (*N* = 1,511) provided survey data on demographics, attitudes, and preferences, and indicated their reactions to 56 social media posts spanning entertainment/lifestyle and news/politics domains with positive and negative framings. Reactions were: *like* or *dislike*,* comment*,* share*, or *no reaction*. Humans could either react or not. If they chose to react, they could like or dislike the post and/or indicate that they would share it and/or comment on it. Survey responses were translated into persona descriptions at three levels of specificity: demographics only, attitudes only, and demographics combined with attitudes. These personas were paired with 27 LLMs, generating 81 agent versions per participant. Each agent predicted the same reactions to the same posts that the corresponding human evaluated. Agent-human agreement was assessed using Hamming accuracy. Prediction accuracy was calculated using hierarchical linear mixed-effects models with participant as a random intercept and LLM type, prompt type, post type, and post valence as fixed effects. Figure 1 shows a complete overview of the process. The dataset can be found at the Open Science Framework (OSF^36^.

### Participants and procedure

Participant recruitment was conducted through Latenta, a market research agency, using Alchemer as the survey aggregation platform. Participants were recruited to provide a broad adult Serbian sample rather than a narrowly defined clinical, professional, or platform-specific population. The final analytic sample was obtained after applying predefined exclusion and quality-control criteria, including removal of incomplete and low-quality responses. Thus, participant validation consisted of survey-completion checks, screening criteria, and post-hoc data-cleaning procedures before agent personas were generated. Data collection took place between November 21 and November 28, 2025. During this period, a total of 3,119 survey attempts were recorded, of which 1,626 were fully completed, 899 were partially completed, and 594 participants were disqualified based on predefined screening criteria. Following data cleaning, during which 115 responses were removed due to issues such as incomplete data or low-quality responses, the final analytic sample consisted of 1,511 participants (50.6% female, 0.2% other), aged 18–78 years (*M* = 36.77, *SD* = 13.46). Most participants had completed high school (36.0%) or university education (15.5%), followed by vocational education (12.6%), while 11.9% were students. The remaining participants reported educational levels ranging from incomplete elementary education to a doctoral degree. A majority of participants were employed (59.4%), with the remainder being unemployed (19.6%), working part-time or freelance (11.2%), retired (4.4%), or reporting another employment status (5.5%). Participants were relatively evenly distributed across Serbian regions: Belgrade (25.5%), Southern and Eastern Serbia (21.1%), Šumadija and Western Serbia (25.9%), and Vojvodina (27.7%).

Participants accessed the survey online and first completed basic demographic information. They were then presented with posts and asked to indicate their possible reactions: *like* or *dislike*,* comment*,* share*, or *no reaction*. If the option *no reaction* was selected, no other reaction could be chosen. Since participants could choose either *like* or *dislike* (but not both), and provided that *no reaction* was not selected, the total number of reactions per post could range from one to three. After responding to the posts, participants proceeded to complete the remaining survey items.

### Measure

The survey consisted of two main parts: social media posts and self-report items. The posts were constructed as benchmarking stimuli rather than as a psychometrically validated attitude scale. They were designed to systematically cover the main experimental dimensions of interest: content domain, emotional valence, and whether the post topic was related or unrelated to available persona information. This design allowed the same prediction task to be evaluated across comparable types of social media content. The social media posts were written in Serbian, in a first-person social media voice. In total, 56 posts were created, including both positive (*n* = 31) and negative framings (*n* = 25). The posts were equally divided between two content categories: news/politics and entertainment/lifestyle, as well as two categories referring to their connection with self-report items: unrelated-unmatched posts and related-matched posts. News/politics posts covered topics such as trust in institutions, geopolitical positions, and domestic political issues, while entertainment/lifestyle posts addressed everyday preferences and activities including media consumption, hobbies, and cultural tastes. Related posts corresponded directly to self-report items that the participant answered after posts, meaning the agent persona derived from that participant’s data contained relevant attitudinal information about the post topic. Persona construction followed a deterministic rule-based procedure: demographic personas contained only basic sociodemographic variables; attitude personas contained survey-derived attitudes, preferences, habits, and personality-relevant information; and full personas combined all available information. Persona validation therefore focused on structural consistency across participants and prompt conditions, so that each persona level contained the intended information type and that no unavailable or unmatched information was added to the prompt. Unrelated posts had no corresponding items, so no topic-specific attitudinal information was available for persona construction. The task for participants was that for each post indicate a reaction by selecting like or dislike, comment, share, or no reaction, under the structural constraints - like and dislike were mutually exclusive, and selecting no reaction precluded all other choices.

The self-report items refers to basic sociodemographic information (age, gender, education, employment status, and region of residence) alongside a series of items covering various attitudes and behaviors, such as institutional trust (toward the army, healthcare system, media, science, government, police, church, NGOs, and trade unions), political orientation and geopolitical attitudes (toward the Russia-Ukraine conflict, EU integration, Trump, and China), attitudes toward conspiracy theories and ecology, personality traits, leisure time preferences (e.g., reading, gaming), music genre preferences (e.g., classical, pop), and engagement in daily activities such as cooking, shopping, volunteering, interior design, and the use of chatGPT. These items served as the basis for constructing participant-specific agent personas in the subsequent simulation phase.

Participants were divided into two groups, which received mirror-image assignments: posts that were related to self-report items for the first group were unrelated to self-report items for the second group, and vice versa. This counterbalanced design allowed control of potential confounding conditions regarding the posts content.

### LLM agents generation

For each of the 1,511 participants, LLM-based agents were generated by translating survey responses into structured natural-language persona descriptions. Three levels of persona specificity were constructed to test the effect of persona detail on reaction accuracy. The first level included only basic sociodemographic information: age, gender, education, employment status, and region of residence. The second level added information on attitudes, personality characteristics, habits, leisure time activities, trust in institutions. All those were derived from the participant’s survey responses on items thematically relevant to the post stimuli. The third and most detailed level incorporated the full set of available survey responses, combining demographics and additional information in a comprehensive persona profile.

Each agent was presented with the same posts that the corresponding human participant evaluated, and the agent’s task was identical to the human task. Reactions were elicited through a standardized prompt that presented the persona description, followed by the post text, and asked the agent to respond as the described person would on a social media platform. In Study 1, the prompt offered all five response categories as a fixed, closed set: like, dislike, comment, share, and no reaction. “No reaction” was an explicit option corresponding to a person who would view the post without engaging, rather than a refusal to answer, and selecting it precluded all other reactions, exactly as in the human task. Each agent had to return a valid selection from this set, and empty or malformed outputs were re-queried rather than counted as non-responses. In Study 2, the response set was reduced to a binary like-or-dislike choice. Full prompt templates for both studies are provided in the Supplementary Appendix and the OSF repository^36^.

A total of 27 large language models were used to generate agent reactions, drawn from nine model families: OpenAI (gpt-5.2-chat-latest, openai/gpt-5.0-chat-latest, GPT-4o mini, GPT-4.1 mini, GPT-OSS-20B), Google (Gemini 2.5 Flash, Gemini 2.5 Flash Lite, Gemini 3 Flash Preview, Gemma 3 27B), xAI (Grok 4.1 Fast, Grok 3 Mini Fast), Mistral (Devstral 2512, Mistral Small 3.1, Mistral Small Latest, Mistral Large Latest), Alibaba/Qwen (Qwen Flash, Qwen Turbo, Qwen Plus), NVIDIA (Nemotron 3 Nano 30B, Nemotron Nano 12B V2), Meta (LLaMA 3.3 70B), Anthropic (Claude Haiku 4.5), DeepSeek (DeepSeek-V3.2), KwaiPilot (KAT Coder Pro), and two additional models from the Nex-AGI and Nous Research families. Models were further classified by inference mode: 17 employed chain-of-thought or extended reasoning, while the remaining 10 operated in standard non-reasoning mode. All models were queried via API under identical prompt structures to allow direct cross-model comparison of behavioral prediction accuracy.

API calls were implemented per provider using analogous batch scripts that shared this prompt structure and a common output-validation logic. Decoding parameters such as temperature, top_p, and max_tokens were not manually specified in the scripts, so provider-default settings were used unless otherwise imposed by the API. Model outputs were requested as structured CSV responses and were automatically parsed and validated according to predefined rules, including the expected number of rows, valid binary values, mutual exclusivity of like/dislike, and consistency of the no-reaction label. Invalid or empty responses were re-queried up to five times with stricter formatting instructions, but these retries were used only to obtain a valid output format and were not treated as repeated experimental runs.

Each model-persona-post condition was processed once for each persona-detail prompt version. The three prompt versions represented different persona-specificity conditions rather than repeated stochastic runs of the same prompt. Because repeated runs were not conducted, temporal stability across repeated API calls, provider-side model updates, and model-version changes was not assessed and is acknowledged as a limitation.

Each participant thus generated 81 agent versions in total, reflecting the crossing of three persona specificity levels with 27 LLMs, yielding over 120,000 unique agent-persona combinations across the full dataset. When expanded across all 56 posts and five reaction labels, the full simulation produced approximately 6.85 million individual reaction observations.

### Data analysis

To establish baselines against which agent accuracy could be evaluated, we employed two approaches. One prompted LLM agents to respond to all 56 posts without any persona information, receiving only the post text and response instructions, and the resulting accuracy of approximately 50% confirmed that LLMs without persona information perform at chance level, lacking an implicit model of typical human responses. The other calculated the majority-class baseline (always predicting the most frequent reaction) and the marginal distribution baseline (randomly sampling according to observed frequencies) from the human response data. These baselines revealed that raw accuracy comparisons can be misleading, since in Study 2, where 69.7% of human responses were like, a trivial always-like strategy would achieve 69.7% accuracy. We therefore adopted chance-corrected metrics, particularly MCC, balanced accuracy, and minority-class lift, as primary indicators of genuine predictive validity in Study 2, while reporting raw accuracy alongside these metrics for completeness.

Hamming accuracy was calculated for agents’ data as the proportion of correct matches between agents’ and human reactions, again, for each reaction and on average level. Although *like* and *dislike* were mutually exclusive response options at the behavioural level, in the present analyses they represent separate accuracy indicators since they are not complementing each other because of the possibility of presence of other reactions (i.e., not selected either *like* or *dislike*).

The main analysis applied a hierarchical linear mixed-effects modelling approach, with LLM, prompt type, and post type specified as fixed effects and participant ID, on which the agents were based, included as a random intercept. Model parameters were estimated using restricted maximum likelihood (REML) because it provides less biased estimates of random-effect variance components and, in turn, reliable standard errors for fixed effects in mixed-effects models with random intercepts^37^. Given the focus on accurate estimation and interpretation of model parameters, REML was used for reporting fixed-effect estimates and conducting post hoc comparisons, and post hoc pairwise comparisons between levels of fixed factors were conducted using Tukey-adjusted estimated marginal means. Effect sizes for pairwise comparisons were expressed as standardized mean differences (Cohen’s d), calculated from model-based estimated marginal means and standardized by the residual standard deviation of the mixed-effects model rather than from raw group means, to maintain consistency with the multilevel modeling framework^38^. Following conventional guidelines, d ≈ 0.20 indicates a small effect, d ≈ 0.50 a medium effect, and d ≈ 0.80 a large effect. Model comparisons between nested models differing in fixed effects were conducted separately using likelihood ratio tests based on maximum likelihood estimation (ML), in line with recommended practice, because REML likelihoods are not comparable across models with different fixed effects.

The analyses were conducted in R^39^ using the following packages: *lme4* for fitting linear mixed-effects models^40^, *lmerTest* for obtaining p-values for fixed effects using Satterthwaite’s approximation^41^, *emmeans* for estimating marginal means and conducting Tukey-adjusted pairwise comparisons^42^, and performance for computing marginal and conditional *R*^2^ and related model diagnostics^43^.

### Baseline specifications and evaluation metrics

Agent performance was evaluated against multiple baselines representing different levels of predictive sophistication. The random chance baseline (50% for binary classification) represents prediction with no information. The no-persona LLM baseline consists of LLM responses to posts without any persona description, testing whether persona information contributes to prediction, and this baseline achieved approximately 50% accuracy, indicating that LLMs lack an implicit “default human” response model. The marginal distribution baseline represents expected accuracy from randomly sampling reactions according to their population frequencies (57.8% for binary like/dislike classification in Study 2), capturing prediction that matches aggregate patterns without individual conditioning. The majority-class baseline represents accuracy from always predicting the most common reaction (69.7% for Study 2, given that 69.7% of human responses were “like”), representing the ceiling for any strategy that does not capture individual deviations from typical behavior.

Because raw accuracy can be misleading when classes are imbalanced, we additionally report chance-corrected metrics. The Matthews Correlation Coefficient (MCC) ranges from − 1 to + 1, with 0 indicating no predictive relationship.

Balanced accuracy averages sensitivity across classes, weighting minority and majority classes equally. Minority-class lift measures the ratio of precision to base rate for underrepresented reactions (e.g., dislike), quantifying how much better than random guessing the agent performs for detecting atypical behaviors. These metrics collectively distinguish between population-level pattern matching and individual-level behavioral prediction.

**Fig. 2:**
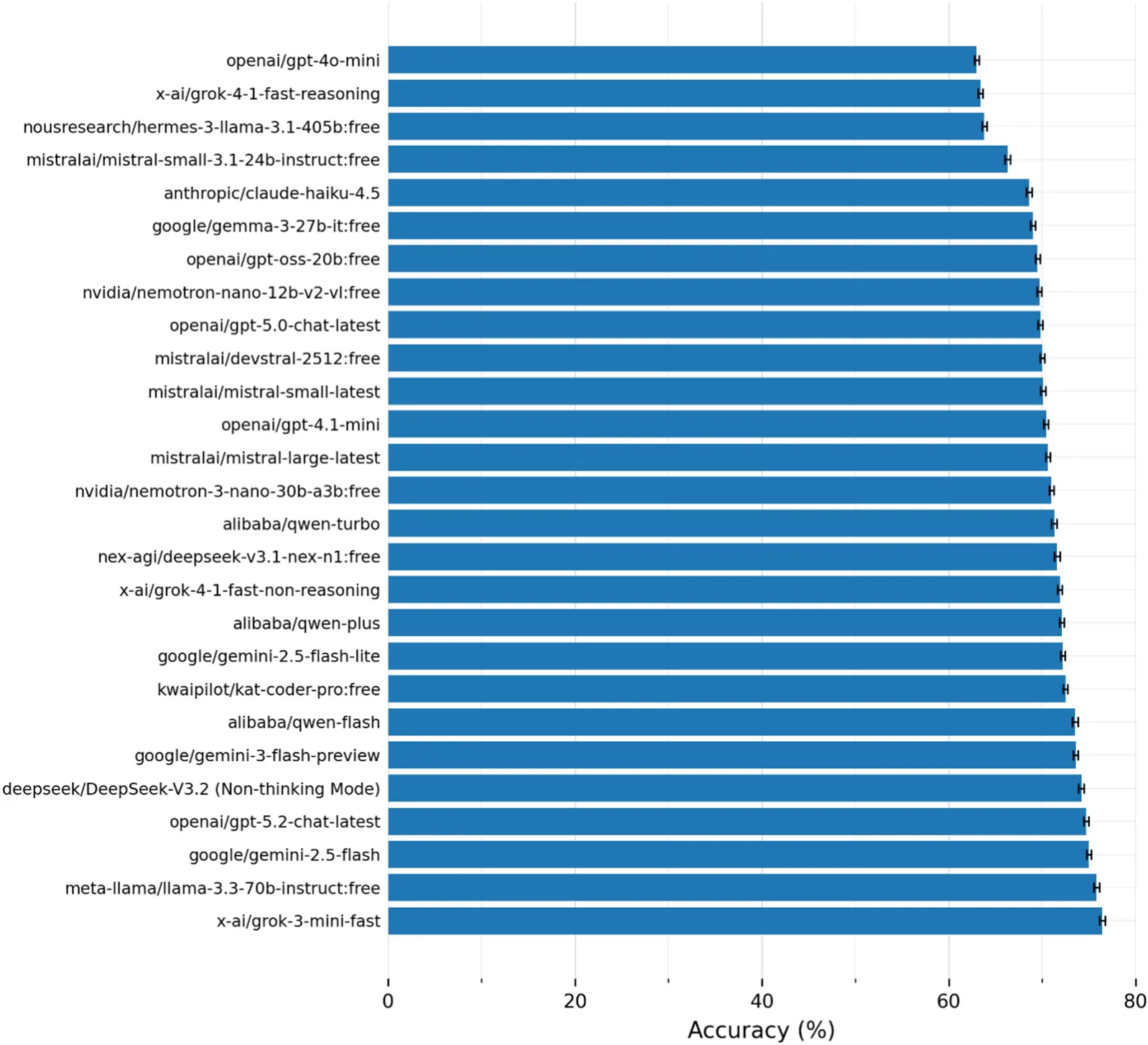
Prediction accuracy by language model (Study 1). Estimated marginal mean accuracy in reproducing human reactions is shown for each of the 27 LLMs. Accuracy ranged from 62.9% to 76.3%, a spread of roughly 13% points, identifying model choice as the strongest single predictor of performance, and the best-performing models were x-ai/grok-3-mini-fast, meta-llama/llama-3.3-70b-instruct, and google/gemini-2.5-flash.

**Fig. 3:**
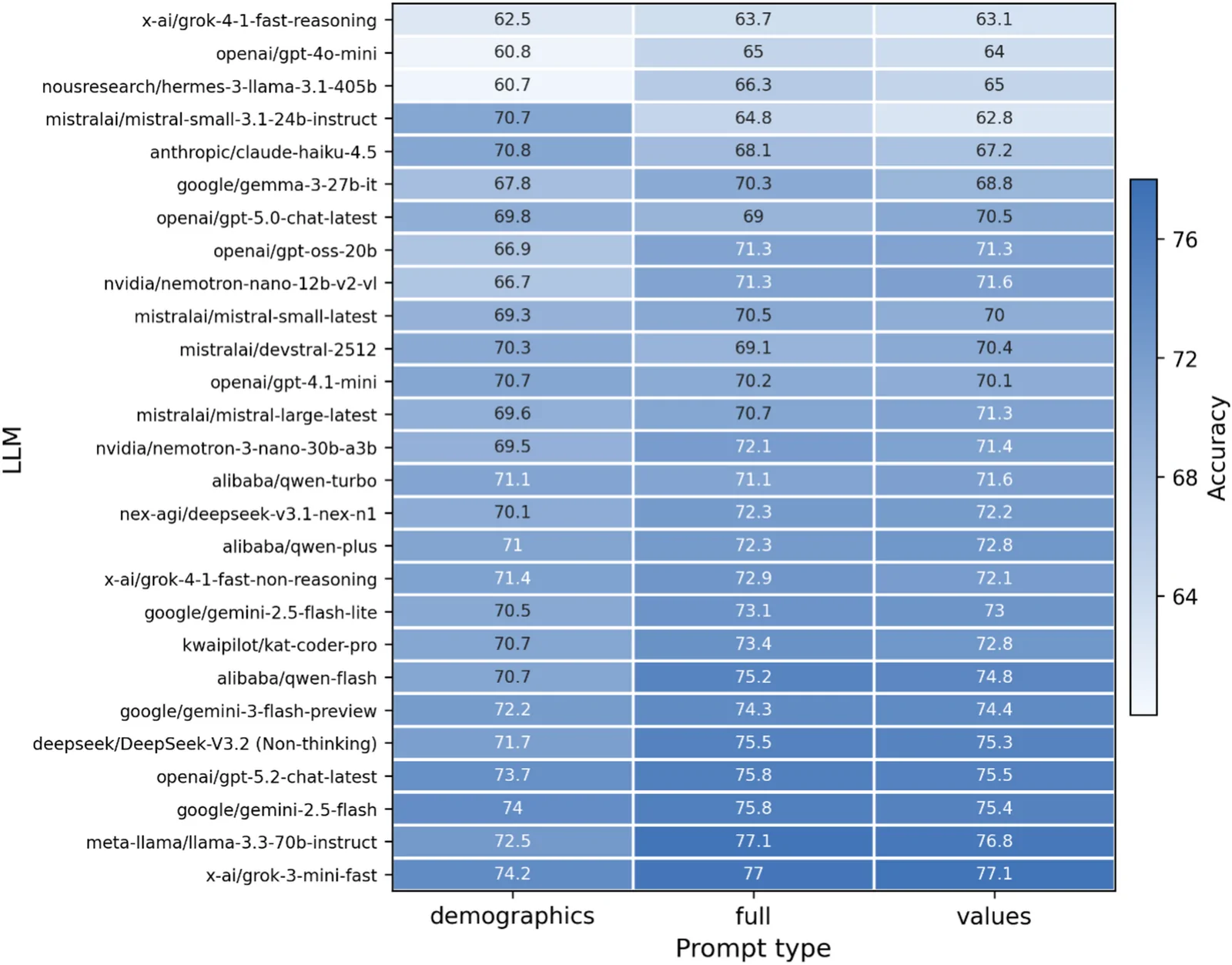
Prediction accuracy by LLM across the three persona-specificity conditions (demographics only, values, and full persona descriptions). Richer personas produced small and inconsistent gains over demographics alone (full 71.5%, values 71.2%, demographics 69.6% on average), indicating that added attitudinal detail contributed little beyond basic demographic anchoring.

**Fig. 4:**
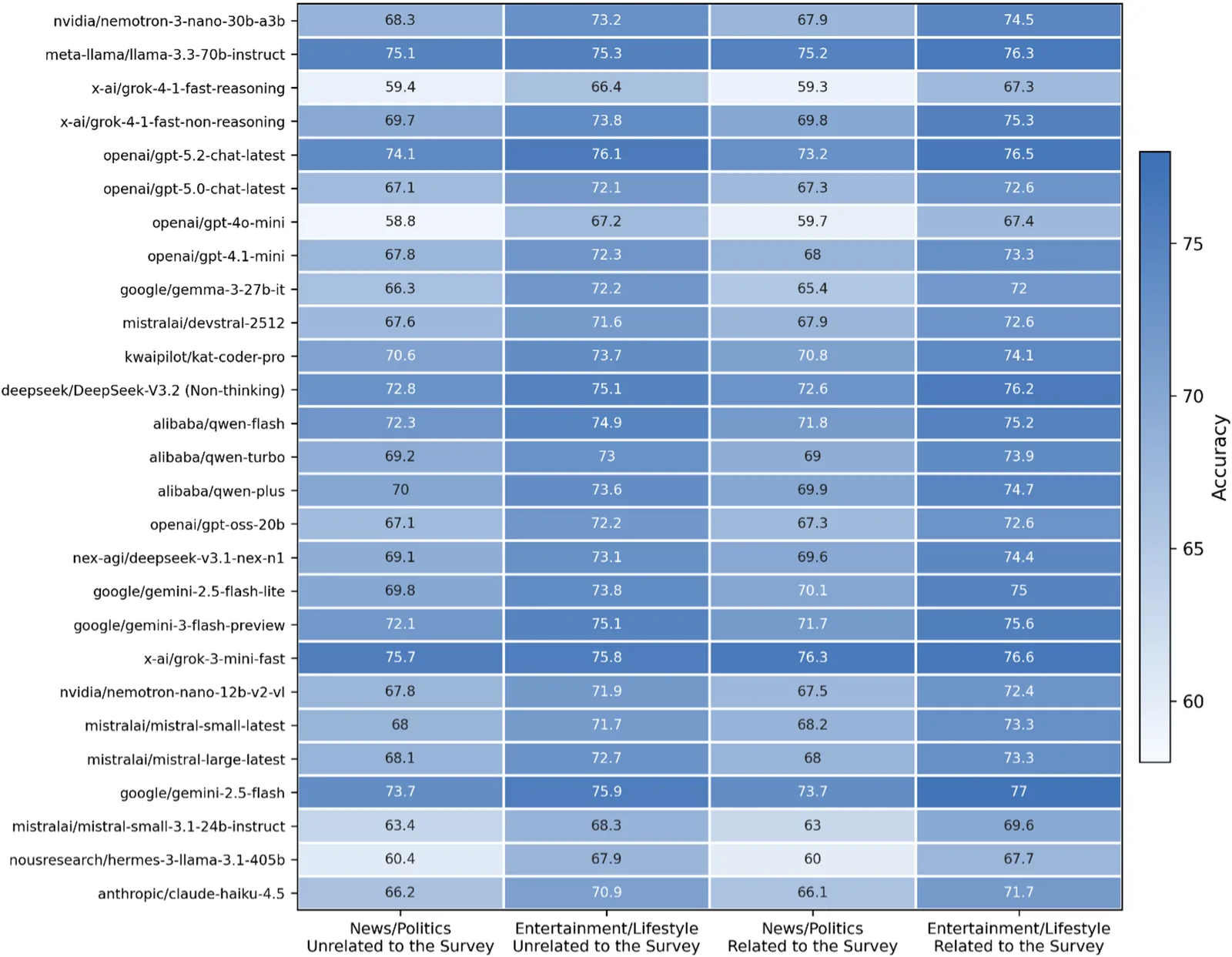
Prediction accuracy across the 27 LLMs by post type and persona-content relatedness. Entertainment/lifestyle posts were predicted more accurately than news/politics posts (roughly 73% versus 68.5%), whereas matching the persona to the post topic produced only trivial and inconsistent gains, a pattern that held across nearly all models.

**Fig. 5:**
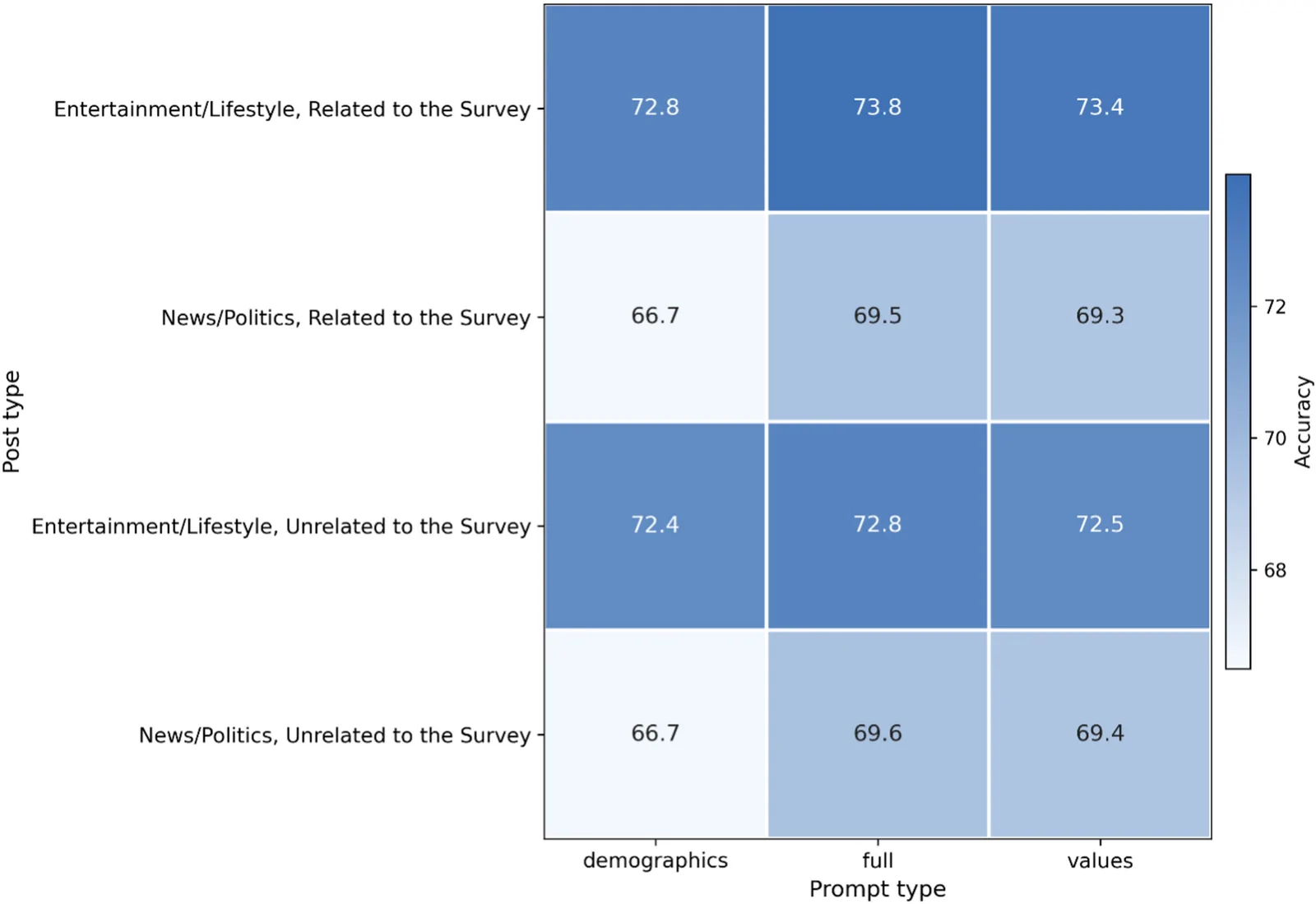
Interaction between post type and prompt specificity. The entertainment/lifestyle advantage over news/politics was present at every prompt level, and richer prompts raised accuracy slightly more for news/politics posts than for entertainment/lifestyle posts, consistent with attitudinal detail mattering most where individual political stances diverge.

To clarify the relationship between accuracy metrics across the two studies: Study 1 uses Hamming accuracy, defined as the proportion of correct matches across all five reaction labels for each agent-post pair. Because Hamming accuracy treats each label independently and most labels have high base rates of non-occurrence (e.g., only 2.78% of cases involve sharing), this metric can yield high values even when agents fail to predict the more informative and rarer reactions. Hamming accuracy is therefore useful for characterizing overall agreement patterns but is a coarse measure that can overstate predictive performance. The prediction accuracy shown in Figs. 2, 3, 4 and 5 is the outcome variable in the mixed-effects models. These models break accuracy down by experimental condition (LLM, prompt type, post type, and valence) to reveal the systematic sources of variation. Study 2 adopts MCC as the primary metric because it equals zero for any strategy that ignores individual-level information, providing the most rigorous test of genuine predictive signal. We regard MCC as the most informative metric in this study because it is insensitive to class imbalance and directly addresses whether agents predict individual deviations from population base rates.

### Benchmark comparison design

To evaluate whether LLM agents provide unique predictive advantages, we compared agent performance against a suite of conventional supervised machine-learning benchmarks using a within-person held-out-post evaluation framework. It is important to note that the two approaches operate under different information conditions. The LLM-based agent simulations were conducted in a fully zero-shot setting: for each respondent-post pair in the evaluation set, the model received only the respondent’s persona information and the content of the post, was not fine-tuned on the study data, and was not given that respondent’s observed prior likes or dislikes. The supervised benchmark models, by contrast, were trained on a predefined within-respondent training subset of respondent-post pairs and could therefore exploit observed response history from the same respondent, including respondent-level and respondent-by-content-type historical preference rates computed solely from the training subset. The comparison thus represents a deliberately stronger, personalized baseline rather than a strictly information-identical contrast with the zero-shot agents. The split was not k-fold cross-validation. Instead, a fixed within-person train-test split was used, with a subset of each respondent’s observed post responses assigned to training and the remaining respondent-post pairs held out for evaluation. All pre-processing, including TF-IDF vectorization, category encoding, imputation, and construction of respondent-specific historical-rate features, was fitted on the training subset only and then applied to the held-out set, preventing test-label leakage. H6 should therefore be interpreted narrowly. It does not claim that the agents and supervised classifiers receive identical behavioral-history inputs, but tests whether zero-shot agent-based simulation, using persona and post semantics alone, can produce useful predictions relative to supervised feature-based models, including stronger personalized models that additionally benefit from observed respondent response histories.

The benchmark suite comprised three families. The structured within-person baselines used respondent-level tabular features (age, gender, region, education, employment status) together with behavioral-history summaries (respondent-specific liking rates) derived from training data. These included a random forest (within_rf_adapt), histogram-based gradient boosting (within_hgb_adapt), logistic regression with adaptation features (within_logit_adapt_plus), and a minimal persona-only logistic regression (persona_only_logit).

Text-based supervised benchmarks tested whether standard classifiers could exploit semantic content directly. Respondent attributes were concatenated into short persona texts, and posts were represented as text combining original content with descriptive annotations. TF-IDF vectorization was applied to both persona and post text, and the concatenated features were used in class-balanced logistic regression. Two variants were tested: text_persona_post_tfidf (text features only) and text_plus_history_tfidf (text features plus behavioral-history summaries). The LLM-agent benchmarks, corresponding to the four best-performing models from Study 1, used prompt-based inference, conditioning large language models on persona descriptions and post text to simulate respondent reactions.

This hierarchical structure distinguishes three possibilities: that predictive performance arises primarily from structured features and behavioral regularities, that performance improves with semantic access to persona and post content, or that LLM agents provide advantages beyond conventional supervised learning.

## Results

### Study 1

Based on the total possible reactions on all posts, participants mostly liked posts or had no reaction, whereas dislike, comment, and share occurred substantially lower (Table 1). The largest discrepancy relative to participants was observed for dislike, which agents produced substantially more often, while no reaction was markedly underproduced.

Study 1 served as an exploratory investigation to identify which LLMs, prompt types, and content characteristics were associated with higher prediction accuracy across five reaction types. Because the multi-label classification structure and class imbalance complicate the interpretation of accuracy metrics (e.g., predicting no share is correct 97.2% of the time due to base rates), Study 1 results should be interpreted as identifying relative performance differences across conditions rather than as evidence of absolute predictive validity.

**Table 1:** Distribution of reaction types for humans and LLM agents across all posts.

Reaction types	Human data		Agents data		Agents accuracy	
	f	%	f	%	f	%
No reaction	25,648	30.31	1,167,652	17.04	4,100,667	59.83
Like	37,128	43.88	3,123,281	45.57	3,825,322	55.81
Dislike	16,632	19.66	2,435,675	35.54	4,217,160	61.53
Comment	5768	6.82	808,380	11.79	5,703,994	83.22
Share	2352	2.78	262,641	3.83	6,383,091	93.13

First, we checked whether there were differences between groups in Hamming accuracy. Results showed significant differences between the two groups, Welch’s *t*(1,022,434) = 29.90, *p* < 0.001. Agents in the first group showed slightly higher accuracy (*M* = 0.71, *SD* = 0.20) than in the second group (*M* = 0.70, *SD* = 0.20), with a mean difference of approximately 0.012. However, the magnitude of this difference was negligible (Cohen’s *d* = 0.06), indicating that despite statistical significance driven by the very large sample size, the practical relevance of the group difference was minimal. Cohen’s *d* for each reaction was in range from 0.01 to 0.09. Thus, we combined scores from the both groups in further analyses. Second, we calculated accuracy for all reactions and it ranged from 55.81% for *like* to 93.13% for *share*, with average across reactions was 70.70%.

Raw accuracy exceeded the no-persona baseline of 50% across all reaction types, confirming that persona information contributes to prediction. However, interpretation of these accuracy values requires caution: the majority-class baseline for this multi-label task is approximately 79.3% (achieved by always predicting the absence of each reaction), which the agents did not exceed. This pattern suggests that agents learned to approximate population-level response distributions rather than to predict individual-level deviations, a question we address more rigorously with chance-corrected metrics in Study 2.

The distribution of Hamming accuracy across all agent-post pairs was unimodal and centered around 0.60, indicating that agents typically matched three out of five reaction labels correctly. Complete agreement with human responses (accuracy of 1.00) occurred in 28.18% of cases. Among partial matches, combinations centered on comment and share dominated: correct prediction of dislike, comment, and share accounted for 19.07% of cases, while like, comment, and share and comment, share, and no reaction each occurred in approximately 15% of cases. Single-reaction matches were rare (below 1%). This pattern indicates that agents most reliably captured commenting and sharing behavior, while combinations involving dislike were comparatively uncommon.

The results of the hierarchical linear mixed-effects models indicated that across models, the Intraclass Correlation Coefficient (ICC) remained stable at approximately 0.03, indicating that about 3% of the total variance in accuracy was attributable to stable between-individual differences captured by the random intercept (Table 2). However, after the inclusion of post type, the ICC increased to 0.09, suggesting that accounting for post characteristics altered the partitioning of variance and revealed stronger clustering at the individual level. With the addition of post evaluation in the final model, the ICC decreased again to approximately 0.03, indicating that this variable accounted for a substantial portion of the variance previously attributed to between-individual differences. Although marginal and conditional *R*² values increased only modestly across tested models, the sequential inclusion of LLM (additional 3%), prompt type (additional 0.2%), post type (additional 1%), and post evaluation (additional 1.3%) led to significant improvements in model fit. This pattern suggests that these predictors exert systematic but relatively small effects on accuracy, with the dominant contribution attributable to LLM choice.

**Table 2:** Model comparison for predicting LLM agents accuracy.

Model	Fixed effects	AIC (ML)	BIC (ML)	Δχ² (df)	Marginal *R*²	Conditional *R*²	ICC
M0	Intercept	-2,602,843	-2,602,802		0.000	0.028	0.028
M1	+ LLM	-2,817,230	-2,816,832	214,439 (26)***	0.030	0.058	0.029
M2	+ prompt type	-2,829,864	-2,829,438	12,637 (2)***	0.032	0.060	0.029
M3	+ post type	-2,906,067	-2,905,599	76,209 (3)***	0.0042	0.070	0.0090
M4	+ post evaluation	-3,000,395	-2,999,914	94,330 (1)***	0.055	0.083	0.028

***p < 0.001.

Regarding LLM, estimated marginal means from the final model (M4) indicated that accuracy ranged from 62.9% to 76.3% across models (Fig. 2). The highest accuracy was achieved by x-ai/grok-3-mini-fast (76.3%), followed by meta-llama/llama-3.3-70b-instruct (75.8%), google/gemini-2.5-flash (75.2%), openai/gpt-5.2-chat-latest (74.8%), and deepseek/DeepSeek-V3.2 Non-thinking Mode (74.3%). In contrast, the lowest performance was observed for openai/gpt-4o-mini (62.9%), x-ai/grok-4-1-fast-reasoning (63.3%), and nousresearch/hermes-3-llama-3.1-405b (63.5%). Pairwise comparisons showed that most differences between LLMs were statistically significant (*p* < 0.001), reflecting the very large sample size. However, the magnitude of these differences was generally small: differences among the top-performing models were minimal (typically ≤ 1% point), whereas the gap between the highest- and lowest-performing models reached approximately 13.4% points. Among the top five performing LLMs, pairwise comparisons revealed that x-ai/grok-3-mini-fast achieved the highest accuracy, though differences among the top-ranked models were very small, with effect sizes ranging approximately from d = 0.02 to d = 0.08. Differences between the top-performing LLM and mid-ranked models were small (approximately d = 0.20–0.35), whereas differences became moderate only when comparing the best-performing models with the lowest-performing ones (approximately d = 0.65–0.69).

Regarding prompt type, results showed that the full prompt yielded the highest accuracy (71.5%), followed by the attitude prompt (71.2%), while the demographics prompt showed the lowest accuracy (69.6%). All pairwise differences were statistically significant (*p* < 0.001), but the effect sizes were small (*d* = 0.01–0.10). Although the absolute differences between prompt types were small, these results indicate a consistent advantage of richer prompt formulations for improving LLM agents accuracy, in line with **H2**. See Fig. 3.

Regarding persona-content alignment, the pattern of results was inconsistent across content domains. For entertainment/lifestyle posts, related posts showed marginally higher accuracy (73.3%) than unrelated posts (72.9%), a difference that was statistically significant but trivial in magnitude (*d* = 0.02). For news/politics posts, accuracy was identical for related and unrelated posts (68.5% each). Because persona-content alignment did not consistently improve prediction accuracy across both content domains, **H3** was not supported in Study 1.

Regarding post type, the highest accuracy was observed for entertainment/lifestyle posts related to the self-report items (73.3%), followed by entertainment/lifestyle posts unrelated to the self-report items (72.9%), as seen in Figs. 4 and 5. Substantially lower accuracy was found for news/politics posts, both unrelated (68.5%) and related to the self-report items (68.5%). All pairwise differences were significant (*p* < 0.001), with the difference between related and unrelated news/politics posts was *p* = 0.014. Effect sizes were generally small, with trivial differences between the two entertainment/lifestyle conditions (*d* = 0.02) and between the two news/politics conditions (*d* = 0.003), whereas comparisons between entertainment/lifestyle and news/politics posts showed small effects (*d* ≈ 0.23–0.25). Overall, LLM agents predicted human reactions more accurately for entertainment/lifestyle posts than for news/politics posts. This exploratory benchmarking comparison (H4) indicated higher prediction accuracy for entertainment/lifestyle posts than for news/politics posts.

Regarding post evaluation, accuracy was substantially higher for positively framed posts (73.1%) than for negatively framed posts (68.5%). This difference was statistically significant (*p* < 0.001) and corresponded to a small effect size (*d* = 0.24). This exploratory benchmarking comparison (H5) indicated higher prediction accuracy for positively framed than for negatively framed posts.

Although Hamming accuracy was relatively high (≈ 71%), the proportion of variance explained by the predictors was modest. This apparent discrepancy reflects the fact that accuracy captures the absolute level of performance, whereas R² indexes variability in performance attributable to systematic differences between predictors. Because a relatively high baseline level of accuracy was observed across all LLMs, prompt types, and post characteristics, only limited between-condition variance remained to be explained by the predictors. Consequently, the effects of predictors represent reliable but relatively small deviations around a generally high level of accuracy, with LLM type showing the largest contribution to predictive performance.

The prediction accuracy reported in Figs. 3, 4 and 5 refers to the per-reaction Hamming accuracy used as the dependent variable in the mixed-effects models, decomposed by condition. This measure differs from the aggregate Hamming accuracy reported above in that it isolates the contribution of specific experimental factors (LLM, prompt type, post characteristics) to prediction performance.

### Study 2

Inspection of the reaction-level accuracy results from Study 1 revealed that like and dislike were predicted less accurately than other reaction types. Study 2 was designed to provide rigorous evaluation of predictive validity using chance-corrected metrics in a simplified binary task. Given that like and dislike represent the most prevalent and arguably most consequential engagement signals on social media platforms, this relative underperformance warranted closer examination. To investigate whether constraining the response space would improve valence prediction, a Study 2 was conducted using a focused paradigm in which agents were prompted exclusively to evaluate whether they would like or dislike each post, with no other reaction options available. This binary forced-choice design eliminates the strategic ambiguity present in Study 1, where an agent may have defaulted to dislike rather than no reaction (or vice versa) for reasons unrelated to genuine valence assessment. For computational efficiency while still covering the top of the performance distribution, agents were generated using only the four best-performing LLMs identified in Study 1: x-ai/grok-3-mini-fast, meta-llama/llama-3.3-70b-instruct: free, google/gemini-2.5-flash, and openai/gpt-5.2-chat-latest. Human data were filtered to include only posts that a given participant had responded to with either a like or a dislike (55.85% of the total post data), excluding all observations where the participant selected comment, share, or no reaction. Agent responses were then compared against this human-conditional subsample, allowing a direct and unconfounded assessment of how accurately LLM agents can predict the valence of human social media engagement when valence is the only dimension under evaluation. The additional change in methodology is that instead of three prompt types, only two were used (demographics and full persona descriptions). The decision was based on the results of Study 1, which showed only marginal accuracy differences between the two prompt types that included additional information beyond demographics. The prompt structure therefore remained the same, with the only change appearing in the section that granted autonomy to the agents by asking for a binary reaction to each post in the form of either like or dislike.

### Overall performance and baseline comparisons

Human participants selected like for 69.7% of posts and dislike for 30.3%, establishing substantial class imbalance. Table 3 presents agent performance against multiple baselines and using chance-corrected metrics.

**Table 3:** Agent performance against multiple baselines (Study 2, LLM02: GPT-5.2).

Metric	Value	Interpretation
Overall Accuracy	67.0%	Raw proportion correct
Random Chance Baseline	50.0%	No information used
No-Persona LLM Baseline	~ 50%	LLM without persona
Marginal Distribution Baseline	57.8%	Matches population rates randomly
Majority-Class Baseline	69.7%	Always predicts “like”
**Matthews Correlation Coefficient**	**0.29**	Genuine predictive signal (0 = no signal)
Balanced Accuracy	65.5%	Equal-weighted class performance
Sensitivity (Like)	69.4%	Correctly identifies likers
Sensitivity (Dislike)	61.5%	Correctly identifies dislikers
Precision (Like)	80.5%	When predicting like, usually correct
Precision (Dislike)	46.7%	When predicting dislike, correct ~half
Minority-Class Lift (Dislike)	1.54	54% better than base-rate guessing

The MCC of 0.29 provides the critical test of **H1**. An MCC significantly greater than zero indicates genuine predictive signal that cannot be attributed to the trivial strategies described above. The observed MCC of 0.29 falls in the “fair” range of predictive association^44^ and confirms that persona-prompted LLM agents capture meaningful covariation between persona-post combinations and human reactions. Therefore, **H1** is supported.

The minority-class lift of 1.54 for dislike predictions indicates that when agents predict dislike, they are 54% more likely to be correct than random base-rate guessing would achieve. This demonstrates practical utility for identifying users likely to react negatively, despite the overall positivity bias. Balanced accuracy of 65.5% confirms reasonably consistent performance across both classes, though sensitivity was higher for like (69.4%) than dislike (61.5%), reflecting the positivity bias documented throughout this research.

Hierarchical mixed linear models (Table 4) indicated that LLM type explained 1.3% of variance in accuracy, the largest contribution among predictors. Prompt type, post type, and post valence collectively explained an additional 2% of variance. While explained variance was modest, effects were statistically robust and theoretically interpretable.

**Table 4:** Model comparison for predicting LLM agents accuracy.

Model	Fixed effects	AIC (ML)	BIC (ML)	Δχ² (df)	Marginal *R*²	Conditional *R*²	ICC
M0	Intercept	521,414	521,447		0.000	0.031	0.031
M1	+ LLM	516,312	516,377	5,108.6(3)***	0.013	0.045	0.032
M2	+ prompt type	516,261	516,336	53.0(1)***	0.013	0.045	0.032
M3	+ post type	515,511	515,620	755.2(3)***	0.015	0.046	0.032
M4	+ post evaluation	508,507	508,626	7,006.5(1)***	0.033	0.063	0.031

***p < 0.001.

Regarding LLMs, GPT-5.2 and Gemini-2.5-Flash achieved the highest performance. Estimated marginal means for accuracy indicated that GPT-5.2 (67.8%) and Gemini-2.5-Flash (67.4%) outperformed LLaMA-3.3-70B (56.9%) and Grok-3-mini-fast (56.1%). Effect sizes were small (d = 0.02 to 0.25), but the consistency of the GPT-5.2 and Gemini advantage across metrics suggests these models better capture human reaction patterns.

Tukey-adjusted pairwise comparisons indicated that gemini-2.5-flash and openai/gpt-5.2-chat-latest did not differ significantly (*p* = 0.23), while both models predicted significantly more like reactions than grok-3-mini-fast and llama-3.3-70b (all *p* < 0.001). The difference between grok-3-mini-fast and llama-3.3-70b was also significant (*p* = 0.0028). Effect sizes were small overall, with *d* ranging from 0.02 to 0.25.

Accuracy was marginally higher for full persona prompts (62.6%) compared to demographics-only prompts (61.5%), a statistically significant difference (*p* < 0.001) with negligible effect size (d ≈ 0.02). This minimal difference suggests that, for binary valence prediction, basic demographic information captures most of the persona-relevant signal, with attitudinal details providing limited additional value. Thus, **H2** receives only weak support in Study 2.

Accuracy varied by post type, i.e., persona-content alignment. Entertainment/lifestyle posts were predicted more accurately (63.8% averaged across related/unrelated) than news/politics posts (60.3%), supporting H4. Persona-content alignment improved accuracy specifically for news/politics posts: related news posts (61.9%) were predicted more accurately than unrelated news posts (58.7%), a difference of 3.2% points. For entertainment posts, the alignment effect was smaller (64.4% vs. 63.1%, difference of 1.3 pp). This pattern suggests that specific attitudinal information in the persona is more valuable for predicting reactions to political content, where individual differences in stance are more consequential. Therefore, **H3** receives partial support, with alignment effects concentrated in the political domain.

Post valence produced the largest effect on accuracy. Positive posts were predicted with 67.9% accuracy compared to 54.8% for negative posts, a difference of 13.1% points (*p* < 0.001, d = 0.27). This positivity bias was consistent across LLMs and prompt conditions, indicating a systematic tendency for agents to better approximate human reactions to positive content. The bias likely reflects both training data distributions (positive content may be more frequent in LLM training corpora) and the greater consensus in human reactions to positive versus negative content. Therefore, H5 is supported.

### The best models

**Fig. 6:**
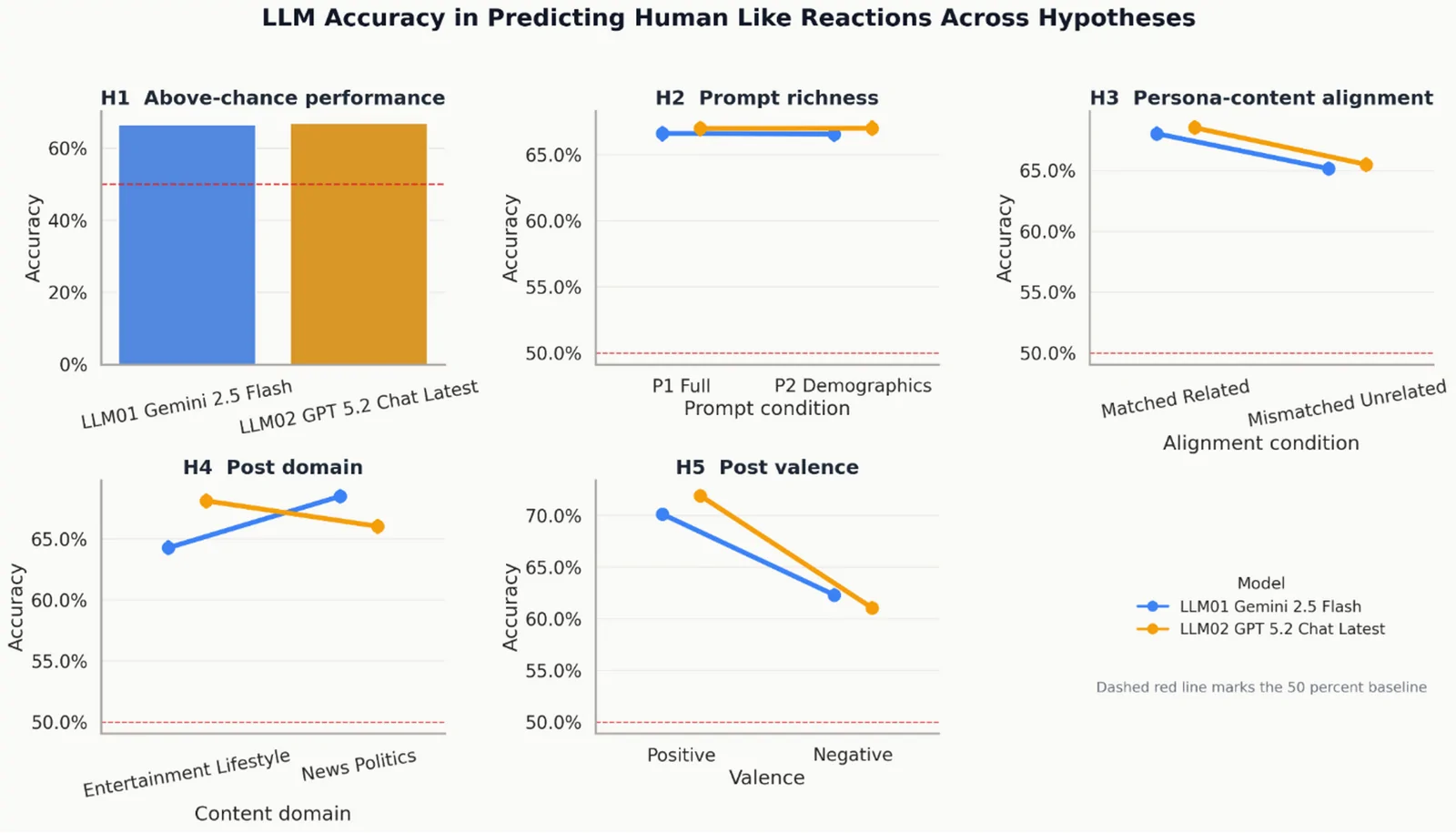
Like-prediction accuracy for the two best models (Gemini 2.5 Flash and GPT-5.2) across hypotheses H1-H5 in the Study 2 binary task. Both models performed above the 50% chance baseline (dashed line) throughout. Accuracy changed little with richer prompts (H2), improved modestly when persona and post were aligned (H3), and was highest for positively valenced posts (H5), where the positivity bias reached up to roughly 11% points, while the domain effect (H4) was small and inconsistent in direction across the two models.

To examine the best models, we focused our inquiry on Gemini 2.5 Flash and GPT 5.2 Chat Latest. The outcome variable was binary accuracy in predicting whether a human participant would register a like reaction (See Fig. 6). Across analyses, both models performed above baseline, but the strength and direction of contextual effects varied by hypothesis.

For **H1**, both models demonstrated genuine predictive signal as assessed by chance-corrected metrics. Gemini 2.5 Flash achieved MCC = 0.29, balanced accuracy = 65.5%, and minority-class lift = 1.54 for dislike predictions. GPT 5.2 achieved comparable performance with comparable MCC. These metrics confirm that both models capture meaningful covariation between persona-post inputs and human reactions that cannot be attributed to majority-class prediction or marginal distribution matching. Raw accuracy (66.6% for Gemini 2.5 Flash, 67.0% for GPT 5.2) fell slightly below the majority-class baseline of 69.7%, but this reflects the models’ attempts to predict minority-class (dislike) reactions rather than a failure of prediction. Therefore, **H1** is supported.

For H2, richer persona prompting did not improve prediction accuracy in the binary task. Both LLMs showed negligible differences between full persona and demographics-only conditions (differences < 0.1 pp, Cohen’s h ≈ 0). This null effect contrasts with the small but consistent advantage of richer prompts in Study 1’s multi-label task, suggesting that demographic information alone is sufficient for binary valence prediction, while finer attitudinal details may contribute to predicting specific engagement behaviors (comment, share). Thus, **H2** was not supported in Study 2.

For **H3**, persona-content alignment improved predictive performance. In Gemini-2.5-Flash, accuracy was 68.05% for matched-related cases and 65.16% for mismatched-unrelated cases, a difference of 2.88% points, 95% CI [2.28, 3.48], with Cohen’s h = 0.061. In GPT-5.2, the corresponding values were 68.54% and 65.51%, a difference of 3.03% points, 95% CI [2.43, 3.63], with Cohen’s h = 0.064. Although the standardized effects were small, they were highly consistent across models and in the predicted direction, providing support for **H3**.

For **H4**, the expected advantage for entertainment and lifestyle content over news and politics was not consistently observed. In Gemini-2.5-Flash, entertainment/lifestyle posts were predicted less accurately than news/politics posts, with accuracies of 64.30% versus 68.52%, respectively, yielding a difference of -4.22% points, 95% CI [-4.83, -3.62], with Cohen’s h = -0.089. In GPT-5.2, the pattern reversed: entertainment/lifestyle posts reached 68.16% accuracy compared with 66.06% for news/politics, a difference of 2.09% points, 95% CI [1.49, 2.70], with Cohen’s h = 0.045. Because the direction of the effect differed across models, **H4** was not supported.

For **H5**, post valence had the clearest comparative effect. In Gemini-2.5-Flash, positive posts were predicted more accurately than negative posts, with accuracies of 70.14% versus 62.32%, a difference of 7.82% points, 95% CI [7.21, 8.42], with Cohen’s h = 0.166. In GPT-5.2, the corresponding accuracies were 71.93% and 61.06%, a difference of 10.87% points, 95% CI [10.27, 11.47], with Cohen’s h = 0.231. This pattern was strong, consistent across models, and substantively larger than the alignment effect, providing strong support for **H5**.

The findings show that both LLMs predict human like reactions reliably above chance, but performance depends more on valence and persona-content alignment than on adding richer persona detail to the prompt. Domain effects were present but not stable across models.

### Benchmark comparison

To test **H6**, we compared the best LLM agents against conventional supervised machine-learning benchmarks on the within-person held-out-post task (Table 5). The results revealed a clear ranking across benchmark families, with text-based supervised models achieving the strongest performance.

The best-performing model overall was text_persona_post_tfidf, a logistic regression classifier operating on TF-IDF representations of persona and post text, which achieved MCC = 0.3601, balanced accuracy = 0.6943, and Brier score = 0.2001. The second-best model was text_plus_history_tfidf (MCC = 0.3521, balanced accuracy = 0.6879, Brier = 0.2024).

The best LLM-agent variant was agent_LLM02 (corresponding to GPT-5.2), which achieved MCC = 0.2958, balanced accuracy = 0.6574, and Brier score = 0.3292. Agent_LLM01 followed with MCC = 0.2777. The remaining agent variants performed substantially worse (agent_LLM04: MCC = 0.1190; agent_LLM03: MCC = 0.0903).

Structured within-person baselines performed worse than both text-based models and the top agent variants. The best structured baseline (within_rf_adapt) achieved MCC = 0.1483 and balanced accuracy = 0.5797.

The text-based benchmark exceeded the best LLM agent by 0.064 MCC, a substantively meaningful margin. The lower Brier scores of the text-based models (0.20 versus 0.33) indicate that the conventional supervised classifiers were both more accurate and better calibrated probabilistically. **H6** was therefore not supported, since the LLM agents did not outperform the text-based benchmarks, although they did outperform the structured tabular baselines.

**Table 5:** Benchmark comparison of LLM agents against conventional supervised machine-learning models on the within-person held-out-post prediction task.

Model	Family	MCC	Balanced Accuracy	Brier
*TF-IDF Logistic Regression (Persona + Post)*	Text benchmark	0.3601	0.6943	0.2001
*TF-IDF Logistic Regression (+ History)*	Text benchmark	0.3521	0.6879	0.2024
*GPT-5.2 Agent*	LLM agent	0.2958	0.6574	0.3292
*Gemini-2.5-Flash Agent*	LLM agent	0.2777	0.6468	0.3327
*Random Forest (Within-Person)*	Structured baseline	0.1483	0.5797	0.2494
*Grok-3-Mini Agent*	LLM agent	0.1190	0.5644	0.4325
*Histogram Gradient Boosting (Within-Person)*	Structured baseline	0.1129	0.5504	0.2459
*Logistic Regression (Within-Person + Features)*	Structured baseline	0.0958	0.5509	0.2785
*LLaMA-3.3-70B Agent*	LLM agent	0.0903	0.5488	0.4439
*Logistic Regression (Persona Only)*	Structured baseline	0.0474	0.5256	0.2478

## Discussion

The results of this research contribute a large-scale empirical benchmark for the behavioral fidelity of LLM-based social media agents, employing chance-corrected metrics that provide rigorous tests of predictive validity. The central finding is that persona-prompted LLM agents achieve genuine but modest predictive signal for human social media reactions (MCC = 0.29), a level that exceeds chance but falls short of high-fidelity individual-level simulation and does not exceed what conventional text-based supervised classifiers achieve on the same task. This finding confirms that agents capture meaningful covariation between persona-post combinations and human reactions, rather than merely reproducing population-level base rates.

However, the magnitude of this predictive signal is modest. Balanced accuracy of 65.5% and minority-class lift of 1.54 indicate that agents perform better than chance at identifying users who will react negatively to content, but the practical magnitude of this advantage is limited. The results fall well short of the individual-level precision that would be required for applications such as targeted content personalization. The gap between raw accuracy (67%) and the majority-class baseline (69.7%) reflects a meaningful pattern: agents sacrifice some overall accuracy by attempting to predict minority-class reactions (dislikes), a strategy that provides genuine informational value even though it reduces headline accuracy figures.

The finding that LLM choice produced the largest effects across both studies, a 13%-point spread in Study 1 and consistent advantages for GPT-5.2 and Gemini-2.5-Flash in Study 2, carries immediate practical implications. Model selection is the most consequential design decision in any social simulation pipeline, outweighing investments in persona construction or content curation. Researchers building agent-based simulations should treat model benchmarking as a prerequisite rather than an afterthought, and should report chance-corrected metrics to enable meaningful cross-study comparisons.

The benchmark comparison against conventional supervised machine-learning models yields one of the most consequential findings for the simulation literature. Conventional TF-IDF classifiers outperformed every LLM-agent variant, with the best text model exceeding the best agent by 0.064 MCC, and three results converge on a single interpretation. Agents clearly beat the structured baselines that had no access to text, which shows that the textual content carries the predictive signal. A simple logistic regression on TF-IDF features then matched or exceeded the agents, which shows that sophisticated language modelling added nothing beyond a basic text representation. The text classifiers were also far better calibrated than the agents, with Brier scores of 0.20 against 0.33, which is consistent with agents pattern-matching on surface features rather than building coherent behavioral models.

The practical consequence is that researchers can obtain equal or better prediction from conventional text classifiers at a small fraction of the computational cost. The theoretical consequence is that the label “agent” becomes misleading when applied to persona-prompted LLMs in prediction settings, because these systems are performing text classification, and their apparent behavioral fidelity reflects the informativeness of the text they are given rather than any emergent behavioral reasoning.

The persona-specificity result challenges a foundational assumption of the agent-simulation literature. Richer persona descriptions that combined demographics, attitudes, and behavioral preferences improved accuracy by roughly 2% points in Study 1, and the gain disappeared in the binary task of Study 2. Genuine individual-level simulation should have rewarded richer information about the person, so the near-absence of any gain suggests that current models lean on coarse demographic categories, population-level priors, and semantic cues from the post itself rather than integrating the specific persona. The competitive performance of the text classifiers reinforces that reading. Several explanations remain open, since agents may process personas through stereotype activation rather than individual reasoning, the attitudinal detail may be largely redundant with demographics, or the like/dislike outcome may be too coarse to reveal benefits that would surface for finer predictions such as comment content. All of them converge on one question with direct consequences for the field, namely how far LLM agents can predict behavior that is individually distinctive rather than stereotypically predictable.

This pattern points to a structural ceiling. When LLMs encode exaggerated group-level representations from training data, as Guilbeault et al.^22^ show for age and gender, persona prompting may activate those representations rather than a genuine individual model, which lets agents predict stereotype-consistent behavior while missing behavior that departs from it. Xie^45^ report a similar limit, with representational fidelity degrading when individual conditioning is sparse. The marginal return on persona detail therefore stays small once basic demographic anchoring is in place, consistent with the limited explanatory power of persona variables reported by Hu et al.^12^, which suggests that prompting alone has an upper bound and that the supervised fine-tuning advocated by Schwager et al.^46^ is a more promising route.

The interaction between persona-post relatedness and post type, observed most clearly in Study 2, is among the more instructive findings. Persona-content alignment mattered substantially for news and political posts but made almost no difference for entertainment content. When an agent had access to the participant’s attitude toward a specific institution or political issue, its ability to predict dislike improved markedly (from 57.0% to 66.0% in the related condition). This asymmetry implies that entertainment reactions are driven by more generic preferences that demographic information alone can approximate, whereas political reactions depend on specific attitudinal commitments that must be explicitly supplied in the persona.

For researchers building social media simulations, this result suggests that persona construction should prioritize domain-specific attitudinal data when the simulation involves political or ideological content, but that simpler demographic profiles may suffice for entertainment and lifestyle domains.

### Methodological implications: chance-corrected metrics in LLM evaluation

This research illustrates why raw accuracy comparisons can be misleading for evaluating LLM-based social simulation, reinforcing the importance of standard evaluation practices for imbalanced classification. In Study 2, the majority-class baseline of 69.7% (achieved by always predicting “like”) exceeded the agents’ raw accuracy of 67%, which might suggest that agents perform worse than a trivial strategy. This interpretation would be incorrect. The MCC of 0.29 and minority-class lift of 1.54 reveal that agents provide genuine predictive value precisely because they attempt to identify the 30% of cases where humans will dislike content, a task that a majority-class strategy entirely ignores.

This pattern has implications for how LLM simulation research should be evaluated. We recommend that future studies report: (1) MCC as the primary indicator of predictive validity, given its insensitivity to class imbalance and base-rate strategies; (2) balanced accuracy to provide equal weighting across classes; (3) minority-class lift to quantify practical value for detecting atypical behaviors; and (4) per-class sensitivity and precision to diagnose systematic biases. Raw accuracy should be reported for completeness but interpreted cautiously, particularly when outcome classes are imbalanced.

### Practical and policy implications

These findings, while limited to a single prediction task with Serbian participants, carry provisional implications that warrant consideration pending replication across populations and platforms. The societal relevance of these results lies not in high prediction accuracy, which was not achieved, but in the combination of modest accuracy with ease of deployment. An MCC of 0.29 represents weak individual-level prediction, but zero-shot persona prompting requires no training data, no model fine-tuning, and minimal technical expertise. The concern is scale and accessibility. An actor who can deploy thousands of persona-prompted agents, each achieving modestly-above-chance prediction, gains aggregate capabilities that may be consequential even when individual predictions are unreliable.

The rapid advancement of both LLM reasoning capabilities^47,48^ and prompting strategies^49^ suggests that simulation accuracy will likely improve.

Schroeder^50^ warned that swarms of collaborative LLM agents represent an emerging threat to democratic discourse, as adversaries could operate thousands of AI personas that adapt to engagement patterns and manufacture synthetic consensus. The present study’s results give empirical substance to this warning: if agents can predict which posts people will like, the same prediction engine can craft content that maximizes engagement and amplifies particular narratives. The positivity bias documented here compounds this concern. Simulations using current LLMs will systematically underestimate negative engagement and backlash, while adversarial actors optimizing content will produce material skewed toward positive engagement rather than critical scrutiny.

These examples illustrate key ethical and policy considerations surrounding both the malicious use of LLM agents and their deployment in social science research. Since LLM agents can shift from measurement tools to influence actors simply by optimizing for engagement, ethical use in social science research should require an explicit account of foreseeable harms and misuse pathways, along with safeguards that match the study’s level of deception and public exposure^51–53^.

EU policy frameworks, including the Digital Services Act and the AI Act, address GenAI as a systemic risk to civic discourse, mandating disclosure requirements and prohibiting manipulative techniques^54^. The governance lag between policy timelines and rapid technological advancement remains a concern.

For platform governance, behavioral prediction models based on LLM agents could serve as stress-testing tools for content recommendation algorithms, allowing designers to evaluate downstream effects on engagement patterns before deployment. The finding that agent accuracy differs by content domain implies that such stress tests would need calibration for specific content ecosystems.

The documented limitations, MCC of 0.29 rather than 0.8+, positivity bias of 13% points, and negligible effects of persona enrichment in binary tasks, counsel against over-reliance on simulation-based governance for individual-level prediction. However, the genuine predictive signal and meaningful minority-class lift suggest that LLM-based simulation may be appropriate for aggregate-level analysis: identifying content likely to generate polarized reactions, stress-testing recommendation algorithms for systematic biases, and modelling population-level engagement dynamics. Policy frameworks should match application scope to demonstrated capability, using LLM simulations for systemic analysis while recognizing their current inadequacy for individual targeting.

Also, large-scale field experiments have found that reducing exposure to like-minded sources on social media does not significantly alter political attitudes^55^, suggesting that simulation accuracy in this domain may matter less than initially assumed. A simulation that underestimates negative engagement and dissent will underpredict backlash, radicalization, and the spread of adversarial content, which are precisely the dynamics that platform governance most needs to anticipate. Until these biases are corrected, any policy framework that relies on LLM-based simulations for pre-deployment testing should treat simulation outputs as lower-bound estimates of negative dynamics rather than as faithful representations of the full range of user behavior.

## Conclusion

This research provides a large-scale benchmark of LLM agent prediction accuracy, applying standard chance-corrected metrics appropriate for imbalanced classification. The results demonstrate that persona-prompted agents achieve genuine but modest predictive validity (MCC = 0.29): they recover part of the individual-level variation in reactions, yet remain well below the accuracy that reliable person-specific simulation would require, and they do not surpass conventional text-based classifiers on the same task.

With respect to specific hypotheses: **H1** was supported, with agents achieving an MCC of 0.29 in Study 2, exceeding the zero-value produced by random guessing, majority-class prediction, and marginal distribution matching, though the practical magnitude of this signal remains modest. The minority-class lift of 1.54 for dislike predictions further confirms that agents provide informational value beyond trivial strategies. **H2** received mixed support, and this null-to-weak effect is theoretically consequential. Richer persona descriptions improved accuracy by approximately 2% points in Study 1 but provided no advantage in Study 2’s binary task. This suggests that demographic information alone captures most persona-relevant signal for valence prediction, challenging the assumption that detailed persona construction improves behavioral simulation fidelity. **H3** was partially supported, with persona-content alignment improving accuracy specifically for political content where individual attitudinal differences are consequential. **H4** received mixed support. Entertainment/lifestyle content was predicted more accurately than news/politics in Study 1, but in Study 2 the direction of this effect differed across the two best models, so the advantage did not hold consistently. **H5** was strongly supported, with a consistent positivity bias of up to 13% points favoring accurate prediction of reactions to positive content. **H6** was not supported: LLM agents did not outperform text-based supervised benchmarks that incorporated semantic content of personas and posts through TF-IDF vectorization, although they substantially outperformed structured tabular baselines lacking semantic access.

Several limitations warrant acknowledgment. The sample was drawn exclusively from Serbia, constraining cross-cultural generalizability.

The findings should be interpreted within the cultural and linguistic context of the study. All participants were recruited from Serbia, and all posts were written in Serbian, which may shape both human reactions and LLM-agent predictions. Social media behavior is also platform-dependent, as norms for liking, disliking, commenting, and sharing may differ across platforms and user communities. Therefore, the present results should be viewed as a benchmark for Serbian-language social media-style stimuli rather than as direct evidence of universal human social media behavior. Future work should test whether the observed patterns replicate across languages, countries, cultures, and platform environments.

A further limitation concerns the nature of the outcome measure. Our target was a self-reported, hypothetical reaction to each post presented in isolation, rather than an observed engagement decision made within a live feed. Real platform behavior is shaped by feed composition and ordering, the visible reactions of others, social pressure, network structure, and the sheer volume of competing content, none of which were present in our task. Participants and agents therefore evaluated each post as a standalone stimulus, which means the benchmark predicts stated hypothetical reactions to decontextualized content rather than behavior emitted under naturalistic platform conditions. This gap likely constrains the achievable prediction ceiling and may also alter the relative difficulty of specific reactions, since sharing and commenting in particular are strongly conditioned by audience and social context that our design removed. The isolation of stimuli was a deliberate methodological choice that eliminates feed-ordering and exposure confounds and permits a controlled comparison across models, prompts, and content types, but it does so at the cost of ecological validity. Whether the patterns observed here, including the model ranking, the positivity bias, and the persona-content alignment effect for political content, persist when reactions are measured as observed in-platform engagement is an open and important question. Future work should validate these agents against behavioral trace data from live or naturalistic social media environments, where feed context, social influence, and network effects are present.

Human response inconsistencies, participants sometimes reacted to posts in ways that contradicted their survey responses, may have attenuated observed agent accuracy. Future work using smaller samples with in-depth interviews may yield cleaner behavioral data. The study tested only survey-derived personas and did not explore behavioral trace data or fine-tuning approaches that might yield stronger effects. In addition, repeated stochastic runs were not conducted, and temporal stability across repeated API calls, provider-side model updates, and model-version changes was not assessed.

Two methodological points merit emphasis. Raw accuracy is misleading under class imbalance, so we encourage reporting chance-corrected metrics (MCC, balanced accuracy, minority-class lift). Benchmarking agents against strong text-based classifiers shows that their genuine but modest signal (MCC = 0.29) reflects semantic pattern-matching on text inputs rather than individualized simulation. For prediction tasks, conventional text classifiers offer comparable or better performance at lower cost. The value of LLM agents, if any, lies in flexibility and ease of deployment rather than accuracy.

Several future directions follow directly from our findings. Because prediction accuracy varied by up to 13% points across LLMs, with model choice being the single strongest predictor of performance, systematic benchmarking of newer and larger models is warranted to establish whether the current capability ceiling reflects a genuine limit or simply the models tested here. As richer persona prompts yielded only marginal gains and vanished entirely in the binary task, fine-tuning on behavioral trace data rather than prompt engineering appears the more promising route to individualized prediction, as Schwager et al.^46^ suggest. Since text-based classifiers outperformed LLM agents while sharing the same semantic inputs, future work should test hybrid architectures that combine the flexibility of agents with the calibration advantages of supervised text models. The positivity bias of up to 13% points was consistent across models and prompt conditions, so targeted work is needed to diagnose whether it originates in training-data distributions or in human response consensus, since correcting it is a prerequisite for using agents to anticipate backlash and adversarial dynamics. Finally, because all stimuli were Serbian, multilingual and cross-platform replication should test whether the persona-content alignment effect, concentrated in political content, generalizes to other languages and cultures. Given the dual-use potential of these capabilities, interdisciplinary collaboration will remain essential to developing governance frameworks that harness the benefits of LLM-based social simulation while mitigating risks of misuse^56–58^.

## Data Availability

The data that support the findings of this study are available from the Open Science Framework (OSF). The dataset is openly accessible at https://osf.io/6f3qd/overview? view_only=bddac5c47ab8426ba6e63424fe0839a2.
